# PIRIN2 suppresses S‐type lignin accumulation in a noncell‐autonomous manner in Arabidopsis xylem elements

**DOI:** 10.1111/nph.16271

**Published:** 2019-11-11

**Authors:** Bo Zhang, Bernadette Sztojka, Sacha Escamez, Ruben Vanholme, Mattias Hedenström, Yin Wang, Halbay Turumtay, András Gorzsás, Wout Boerjan, Hannele Tuominen

**Affiliations:** ^1^ Department of Plant Physiology Umeå Plant Science Centre Umeå University S‐901 87 Umeå Sweden; ^2^ Department of Plant Biotechnology and Bioinformatics Ghent University Technologiepark 71 9052 Ghent Belgium; ^3^ VIB Center for Plant Systems Biology Technologiepark 71 9052 Ghent Belgium; ^4^ Department of Chemistry Umeå University S‐901 87 Umeå Sweden; ^5^ Present address: Umeå Plant science Centre, Department of Forest Genetics and Plant Physiology The Swedish University of Agricultural Sciences 90183 Umeå Sweden

**Keywords:** Arabidopsis, lignification, noncell‐autonomy, PIRIN, xylem vessel element

## Abstract

- *PIRIN* (*PRN*) genes encode cupin domain‐containing proteins that function as transcriptional co‐regulators in humans but that are poorly described in plants. A previous study in xylogenic cell cultures of *Zinnia elegans* suggested a role for a PRN protein in lignification*.* This study aimed to identify the function of Arabidopsis (*Arabidopsis thaliana*) PRN proteins in lignification of xylem tissues.
- Chemical composition of the secondary cell walls was analysed in Arabidopsis stems and/or hypocotyls by pyrolysis–gas chromatography/mass spectrometry, 2D‐nuclear magnetic resonance and phenolic profiling. Secondary cell walls of individual xylem elements were chemotyped by Fourier transform infrared and Raman microspectroscopy.
- Arabidopsis PRN2 suppressed accumulation of S‐type lignin in Arabidopsis stems and hypocotyls. *PRN2* promoter activity and PRN2:GFP fusion protein were localised specifically in cells next to the vessel elements, suggesting a role for PRN2 in noncell‐autonomous lignification of xylem vessels. Accordingly, PRN2 modulated lignin chemistry in the secondary cell walls of the neighbouring vessel elements.
- These results indicate that PRN2 suppresses S‐type lignin accumulation in the neighbourhood of xylem vessels to bestow G‐type enriched lignin composition on the secondary cell walls of the vessel elements. Gene expression analyses suggested that PRN2 function is mediated by regulation of the expression of the lignin‐biosynthetic genes.

## Introduction

Lignin is one of the most abundant biopolymers on Earth, and has been widely studied because of its ecological, physiological and economic importance. In angiosperm species, lignin is mainly composed of guaiacyl (G), syringyl (S), and *p*‐hydroxyphenyl (H) units, which are derived from lignin monomers coniferyl, sinapyl and *p*‐coumaryl alcohol, respectively. The monomers polymerise by oxidative coupling either as a defence mechanism in response to pathogens or as part of a developmental programme such as during vascular development (Boerjan *et al.*, 2003; Bonawitz & Chapple, 2010). The regulation of xylem lignification is known to involve transcriptional control of the monolignol biosynthetic genes by transcription factors as well as by the transcription‐related Mediator protein complex (Zhao & Dixon, 2011; Hussey *et al.*, 2013; Bonawitz *et al.*, 2014).

Interestingly, lignification of xylem tracheary elements continues *post mortem*, that is after cell death. Early experiments by Pickett‐Heaps (1968), Terashima *et al.* (1986) and Terashima & Fukushima (1988) showed that radioactively labelled lignin monomers were incorporated into cell walls of living tracheary elements, and that the incorporation continued even after the death of the cells. Lignin incorporation into dead tracheary elements was supported by experiments in *Zinnia elegans* (Zinnia) *in vitro* xylogenic cell cultures that demonstrated that the total lignin content of the cell cultures continued to increase even though all lignifying tracheary elements were dead (Hosokawa *et al.*, 2001; Pesquet *et al.*, 2013). In the same *in vitro* system, pharmacological experiments revealed that the *post mortem* lignification of the tracheary elements was enabled by the production and transport of both monolignols and reactive oxygen species from the nonlignifying parenchymatic cells (Ros Barceló, 2005; Pesquet *et al.*, 2013). Finally, Smith *et al.* (2013) showed convincing evidence in intact Arabidopsis plants on how the lignification of xylem vessel elements is at least partially accomplished by the function of the cells neighbouring the vessel elements. These findings have resulted in wide acceptance of the action of ʻgood neighbours’ of lignification, whereby cells that normally do not themselves lignify assist in the lignification of their neighbouring cells. Therefore, even though lignification is clearly initiated in a cell‐autonomous manner during the lifetime of the tracheary elements (including vessel elements), it continues *post mortem* in a noncell‐autonomous manner. *Post mortem*, noncell‐autonomous lignification seems to occur also in the xylary fibres of Arabidopsis, while the interfascicular fibres seem to lignify mostly in a cell‐autonomous manner (Smith *et al.*, 2017). Furthermore, living vessel elements act as good neighbours to provide monolignols to adjacent xylary fibre cells (De Meester *et al.*, 2018).

In an earlier study, we combined pharmacology and gene expression analyses in the Zinnia xylogenic cell cultures to identify genes that are potentially involved in the regulation of noncell‐autonomous lignification (Pesquet *et al*., 2013). One such candidate gene encoded a CUPIN domain‐containing PIRIN protein (gi:219988534; Pesquet *et al*., 2013). PIRINs (PRNs) comprise a family of proteins that are conserved between prokaryotic and eukaryotic organisms such as mammals and plants (Wendler *et al*., 1997), although they seem to be functionally diverse (Adams & Jia, 2005). The Arabidopsis PRN1 protein is involved in responses to UV light, blue light and abscisic acid, in seed germination and in seedling development (Lapik & Kaufman, 2003; Warpeha *et al*., 2007; Orozco‐Nunnelly *et al*., 2014), while PRN2 interacts with a protease to regulate the susceptibility to a vascular pathogen (Zhang *et al*., 2014). In humans, the only PRN protein was identified on the basis of its interaction with Nuclear Factor I (Wendler *et al*., 1997), and has since then been demonstrated to act as a transcriptional co‐regulator for several different transcription factors (Dechend *et al*., 1999; Liu *et al*., 2013). In this study, we demonstrate the function of Arabidopsis PRN2 in noncell‐autonomous lignification of xylem tissues, and present data suggesting that the primary function of PRN2 is to facilitate enrichment of G‐type lignin in the vessel elements, possibly through regulation of the expression of the lignin‐biosynthetic genes from within the vessel‐neighbouring xylem elements.

## Materials and Methods

### Plant material and growth conditions

Arabidopsis T‐DNA insertion mutants were obtained from the Nottingham Arabidopsis Stock Centre (NASC) including *prn1* (SALK_006939), *prn2‐1* (SM_3.15394), *prn2‐2* (SALK_079571), *prn3* (SAIL_1243_E02), *prn4‐1* (SALK_138671), *prn4‐2* (SALK_125909) and *prn4‐3* (SALK_100855). The *prn4‐4* (GT_19099) mutant was obtained from Cold Spring Harbor Laboratory (NY, USA). *PRN2*‐overexpressing lines 6 and 13 (overexpression under the control of the 35S promoter), and the pro*PRN2::PRN2:GFP* line have been described in Zhang *et al*. (2014). *PRN2*‐overexpressing line 29 (Supporting Information Fig. S4d) was generated as described by Zhang *et al*. (2014).

Plants were grown in soil in growth chambers under short‐day conditions (8 h : 16 h, light : dark, 21°C : 18°C, 70% relative humidity) for 4–8 wk and then moved to long‐day conditions (16 h : 8 h, light : dark, 21°C : 18°C, 70% relative humidity). When the inflorescence stems reached a height of 50 cm, the 2‐cm bottom part was harvested for different analyses. After that, newly formed stems were constantly removed until the hypocotyls were harvested at indicated times.

### Cloning and plant transformation

For promoter‐GUS constructions, 2198‐, 2308‐, 527‐ and 2427‐bp fragments upstream of the translational start codons of *PRN1*, *PRN2*, *PRN3* and *PRN4*, respectively, were cloned into the pDONR207 vector by Gateway recombination using BP Clonase II (Invitrogen), followed by recombination into pMDC163 destination vectors (Curtis & Grossniklaus, 2003) using LR Clonase II (Invitrogen). To generate *PRN2* complementation lines, a 3838‐bp fragment of the *PRN2* genomic sequence, including the promoter and the coding region was cloned (for primers see Table S1) by Gateway recombination into the pGWB16 vector (Nakagawa *et al*., 2007) and transformed into the *prn2‐1* mutant. To generate the hybrid aspen (*Populus tremula *×* tremuloides*) pro*PttPRN2*:*GUS* construct, a 845‐bp fragment upstream of the translational start of the gene (Potri.002G231900) was cloned into pDONR207 (as above) and recombined into pKGWFS7 (Karimi *et al*., 2002). This binary vector was used for *Agrobacterium*‐mediated transformation of hybrid aspen (*Populus tremula *×* tremuloides*) clone T89.

To create the pro*PttPRN2::PRN2‐RNAi* construct, an inverted repeat of a 300‐bp fragment from the *PttPRN2* coding sequence, targeting the beginning of the coding sequence, was placed downstream of the 845‐bp *PttPRN2* promoter. The entire pro*PttPRN2::PRN2‐RNAi* sequence was synthesised by Invitrogen, then inserted into the modified pKGW + T35S (the 35S promoter was removed) binary vector, which was used for *Agrobacterium*‐mediated transformation of hybrid aspen T89.

### Histochemical GUS staining

For histochemical GUS staining of Arabidopsis, *in vitro* grown seedlings or cross‐sections of tissue samples obtained by hand sectioning from soil‐grown Arabidopsis were used. Vibratome cross‐sections (70 µm) of the stems of hybrid aspen plants were taken 10 cm above the soil level. Samples were fixed in 90% acetone on ice for 30 min followed by incubation at 37°C in a solution containing 1 mM X‐Gluc, 1 mM K_3_Fe(CN)_6_, 1 mM K_4_Fe(CN)_6_ and 0.1% Triton X‐100 in 50 mM sodium phosphate buffer (Na_2_HPO_4_/NaH_2_PO_4_, pH 7.2). The samples were destained in a 70% ethanol solution and then in a 99% ethanol solution. Light microscopy images were acquired using a Zeiss Axioplan II microscope equipped with an AxioCam charge‐coupled device (CCD) camera (Zeiss, Jena, Germany).

### Confocal laser scanning microscopy

Arabidopsis seedlings expressing pro*PRN2::PRN2:GFP* were stained with 10 µg ml^−1^ propidium iodide for 2 h. The stained seedlings were imaged using a Zeiss LSM780 inverted confocal microscope.

### Pyrolysis‐gas chromatography/mass‐spectrometry (Py‐GC/MS)

Freeze‐dried stems and hypocotyls were ball milled (MM400; Retsch, Haan, Germany) at 30 Hz in stainless steel jars (1.5 ml) for 2 min with one ball (7 mm diameter). In total, 60 ± 10 μg of powder was then applied to the pyroliser (PY‐2020iD and AS1020E; FrontierLabs, Koriyama, Japan) mounted on a GC/MS (7890A/5975C; Agilent Technologies AB Sweden, Kista, Sweden). Pyrolysis was conducted at 450°C. The pyrolysate was separated on a capillary column with a length of 30 m, a diameter of 250 μm and a film thickness of 25 μm (J&W DB‐5; Agilent Technologies Sweden AB). The gas chromatography oven temperature programme started at 40°C, followed by a temperature ramp of 32°C min^−1^ to 100°C, 6°C min^−1^ to 118.75°C, 15°C min^−1^ to 250°C, and finally 32°C min^−1^ to 320°C. Total run time was 19 min and full‐scan spectra were recorded in the range of 35−250 *m/z*. Data processing including peak detection, integration, normalisation and identification was carried out as described in Gerber *et al*. (2012). Minimum three biological replicates, each consisting of a pool of three hypocotyls or stems, were analysed.

### Phenolic profiling

For metabolic profiling, Arabidopsis plants were first grown in short‐day photoperiod (21°C, 9 h : 15 h, light : dark) on soil in pots 5.3 cm wide × 5.5 cm high. Plants were moved to a long‐day photoperiod (21°C, 16 h : 8 h, light : dark) after 8 wk to allow bolting. Plants were grown until the stem reached on average 42 cm in height. An 8 cm inflorescence stem section (between 1 and 9 cm, above the rosette) was harvested, frozen in liquid nitrogen and deprived of all side branches, for nine biological replicates. The material was ground while frozen and extracted with 1 ml methanol (high pressure liquid chromatography (HPLC grade)) at 70°C for 10 min. After centrifugation, methanol was evaporated and samples were suspended in 200 µl water : cyclohexane (1 : 1) for extraction. A 15‐µl sample of the aqueous phase was subjected to ultra‐high‐performance liquid chromatography–mass spectrometry (UHPLC–MS) on a Waters Acquity UHPLC system (Waters Corp., Milford, MA, USA) connected to a Synapt HDMS Quadrupole Time‐Of‐Flight (Q‐TOF) mass spectrometer (Micromass, Manchester, UK), basically as previously described (Vanholme *et al*., 2013). In brief, chromatographic separation was performed on a Waters Acquity BEH C18 column (2.1 mm × 150 mm, 1.7 µm), with the mobile phase composed of water containing 1% acetonitrile and 0.1% formic acid (A) and acetonitrile containing 1% water and 0.1% formic acid (B), all v/v. During the gradient elution, a flow rate of 350 µl min^−1^ was applied, with initialisation at time 0 min, 5% B, 30 min, 50% B, and 33 min, 100% B. Negative mode MS‐setting, chromatogram integration and alignment via Progenesis QI software (Waters) were as previously described (Vanholme *et al*., 2013). The integrated peak area of the *m/z* values (peaks) were normalised with the function ‘normalise to total peak intensity’. The list of peak areas resulting from these parameters was imported in a digital spreadsheet (Microsoft Excel) for further analysis. To select peaks for which metabolites were significantly increased or decreased in *prn2* mutants compared with wild‐type plants, the following criteria were used: present in all samples of at least one genotype; average normalised abundance higher than 100 counts in at least one genotype; analysis of variance (ANOVA) *P*‐value < 0.005 on arcsinh transformed values; *t*‐test *P*‐value < 0.01 on arcsinh transformed values; and two‐fold increased/decreased peak area in *prn2* vs wild‐type. Annotation of compounds matching these criteria was based on accurate *m/z* (± 0.02 Da), isotope distribution and tandem MS (MS/MS) similarities according to Morreel *et al*. (2010a,b). For MS/MS acquisition, two biological replicates per treatment were run on the UHPLC–MS in the negative mode.

### Fourier transform infrared (FT‐IR) microspectroscopy

FT*‐*IR microspectroscopic measurements and data analysis were performed as described by Gorzsás *et al*. (2011). Briefly, 20‐μm‐thick transverse sections were cryo‐sectioned from the hypocotyls of 8‐wk‐old plants. Spectra were recorded using 32 scans at a spectral resolution of 4 cm^−1^ on a Bruker Tensor 27 spectrometer equipped with a Hyperion 3000 microscopy accessory (Bruker Optik GmbH, Ettlingen, Germany), including a liquid nitrogen cooled 64 × 64 mercury cadmium telluride focal plane array detector (Bruker Optik). Cell‐specific spectra were extracted and converted to data point tables using OPUS (v.5.0.53−v.7.0.129; Bruker Optik), and baseline corrected (four‐point linear) and total area normalised in the region 900–1850 cm^−1^, using custom‐built scripts programmed within the matlab environment (v.7.0; MathWorks Inc., Natick, MA, USA), as described by Stenlund *et al*. (2008). Multivariate analysis was performed using simca‐p+ (v.13–v.14; Umetrics AB, Umeå, Sweden), on data from three biological replicates with 10 spectra per cell type from each. Orthogonal projections to latent structures discriminant analysis (OPLS‐DA) models were calculated on centred data by fixed number of components (one predictive and two orthogonal for pairwise comparison), using the default settings of simca‐p for OPLS‐DA models. According to Umetrics, a Q2 above 0.4 can be considered as a good model, as long as it is not 2 units below R2.

### Raman microspectroscopy

Here, 20‐µm‐thick transverse sections were cryo‐sectioned from the stems and the hypocotyls of 8‐wk‐old plants. Raman microspectroscopy (mapping) was performed as described by Gorzsás (2017) on a Renishaw InVia Raman spectrometer equipped with a CCD detector (Renishaw plc, Wotton‐under‐Edge, UK), using a 514 nm Ar^+^ ion laser with 10–100% laser power and 1 s irradiation time set in the software (WiRE, v.3.0; Renishaw), in static mode centred at 1300 cm^−1^ (resulting in a spectral region of *c*. 625–1903 cm^−1^ with *c*. 1 cm^−1^ spectral resolution using a 2400 lines grating). A ×50 magnification lens was used, with 2 µm step sizes (XY direction), in normal or high confocal modes, recording images with a minimum of 100 spectra. Raman shifts were calibrated using the built‐in Si standard of the instrument. Cosmic rays were removed and data noise filtered using the chemometrics package of WiRE (v.3.4, Renishaw). Spectra were exported as .txt files and preprocessed using the open‐source MCR‐ALS script (run in matlab environments, v.14a–v.18a, MathWorks), as provided by the Vibrational Spectroscopy Core Facility. Spectra were cut to the 630–1900 cm^−1^ region to remove noise filtering artefacts, and thereafter baseline corrected (using asymmetrical least squares (Eilers, 2004); λ = 100 000 and *P* = 0.001), smoothed (Savitzky‐Golay filter (Savitzky & Golay, 1964); polynomial order = 1 and frame rate = 5) and total area normalised. After preprocessing, spectra were extracted from voxels representing pure vessel–vessel or fibre–fibre cell walls, compiled in a single Microsoft excel file and imported into simca‐p (v.13.0–v.14.0; Umetrics AB). Multivariate models were calculated using Principal Component Analysis (PCA) and the ʻAutofitʼ options in simca‐p to determine the number of significant components.

### 2D‐NMR spectroscopy

2D‐NMR analysis of acetylated cell wall material was performed as described by Hedenström *et al*. (2009) with a few modifications. Here, 200 freeze‐dried and bark‐peeled 8‐wk‐old hypocotyls were ball milled (MM400; Retsch) at 30 Hz in stainless steel jars (1.5 ml) for 2 min with one ball (diameter 7 mm). The resulting hypocotyl powder was prepared for NMR analysis by additional ball milling and acetylation (Hedenström *et al*., 2009). 2D ^13^C–^1^H heteronuclear single quantum coherence (HMQC) spectra (pulse program hmqcetgp) were acquired at 25°C on a Bruker DRX 600 MHz instrument (Bruker Biospin GmbH, Rhenistetten, Germany) equipped with a triple‐resonance cryo‐probe with z*‐*gradients. Forty‐eight transients, 320 t_1_ increments and an interscan delay of 1 s resulted in an experiment duration of *c.* 5 h for each spectrum. Spectra were manually phase‐ and baseline‐corrected using Topspin 2.0 (Bruker Biospin). The residual CHCl_3_ peak was used as an internal reference (δ_C_ 77.0 ppm, δ_H_ 7.27 ppm). Peaks originating from the aromatic rings in the syringyl (S2,6; Fig. 4d,e) and guaiacyl units (G2, Fig. 4d,e) were integrated and the S : G ratio was calculated using the formula S : G = (*I*_S2,6_ × 0.5)/*I*_G2,_ where *I*_S2,6_ and *I*_G2_ are the volume integrals of peaks from S and G subunits, respectively. *I*_S2,6_ is scaled down by a factor 2 because it contains signals from two protons in the aromatic ring.

### Gene expression analyses

Total RNA was isolated from hypocotyls and stems using an RNeasy Plant Mini Kit (Qiagen) according to the manufacturer’s protocol. All samples were treated using an RNase‐free DNase Set (Qiagen) in columns to remove any remaining DNA. cDNA was synthesised by reverse transcription using oligo(dT) and random primers and 1 µg RNA as a template. Semiquantitative RT‐PCRs were performed using 5× diluted cDNA with *Taq* polymerase for 30 cycles. The Arabidopsis *Actin1* (AT2G37620) was used as a reference gene. Quantitative RT‐PCRs (qPCR) were performed using 40× diluted cDNA, iQ SYBR Green Supermix (Bio‐Rad), 5 pmol of each primer, an iQ5 Multicolor Real‐Time PCR Detection System (Bio‐Rad) and the following programme: initial denaturation at 95°C for 3 min followed by 40 cycles of 10 s denaturation at 95°C and 30 s annealing at 55°C. The Arabidopsis *PDF2* (AT1G13320) and *UBQ10* (AT4G05320) genes were selected as housekeeping genes after assessment by geNORM analysis (Vandesompele *et al*., 2002). Both housekeeping genes were included in all qPCR analysis, their geometrical mean was calculated and used in further normalisation steps. Five biological replicates were used for each analysis. Primer sequences are listed in Table S1.

### Statistical analyses

For all comparisons of means between lines/treatments and the wild‐type/control, Welch’s *t*‐tests were performed assuming unequal variance. For multiple pairwise comparisons of all lines/treatments within one and the same experiment, protected (*F*‐test performed at α = 0.05) post‐ANOVA Fisher’s least significant difference (LSD) tests were used.

### Accession numbers

Nucleotide sequence data from this article can be found in the Arabidopsis Genome Initiative under the following accession numbers: *PRN1* (At3g59220), *PRN2* (At2g43120), *PRN3* (At3g59260), *PRN4* (At1g50590), *PAL1* (At2g37040), *C4H* (At2g30490), *4CL1* (At1g51680), *C3H1* (At2g40890), *HCT* (At5g48930), *CCoAOMT1* (At4g34050), *CCR1* (At1g15950), *F5H1* (At4g36220), *COMT1* (At5g54160), *CAD5* (At4g34230), *VND6* (At5g62380), *VND7* (At1g71930), *NST1* (At2g46770), *NST2* (At3g61910), *SND1* (At1g32770), *MYB46* (At5g12870), *MYB83* (At3g08500), *MYB58* (At1g16490), *MYB63* (At1g79180), *MYB85* (At4g22680), *MYB61* (At1g09540), *MYB103* (At1g63910).

## Results

### *PRN* genes show unique expression patterns in Arabidopsis vascular tissues

We demonstrated earlier in xylogenic cell cultures of Zinnia that the expression of a *PRN* gene was associated with tracheary element lignification in a manner suggesting a role in noncell‐autonomous lignification (Pesquet *et al*., 2013). The Arabidopsis genome contains four genes that encode PRN proteins: *PRN1*, *PRN2*, *PRN3* and *PRN4*. Phylogenetic analysis revealed that PRN1, PRN2, PRN3 proteins, as well as the Zinnia PRN protein, are closely related to each other, while PRN4 is somewhat more distantly related to the other PRN proteins (Fig. S1).

**Figure 1:**
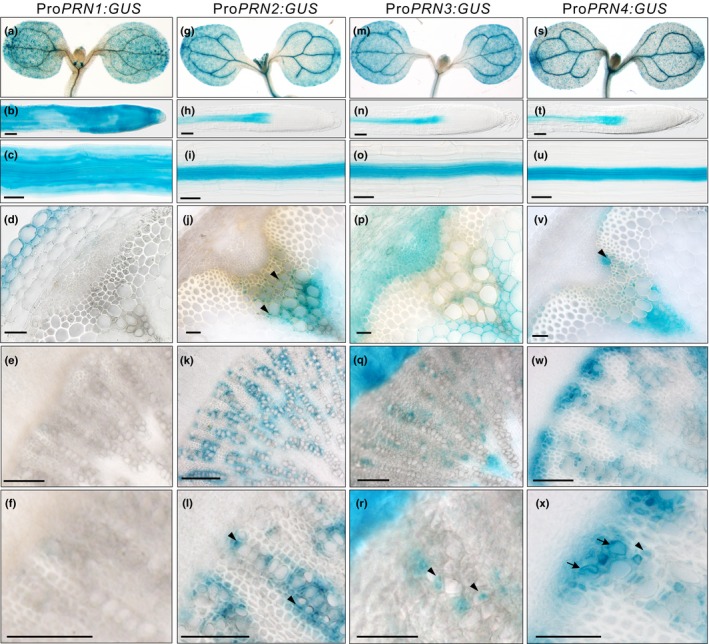
The promoters of the *Arabidopsis thaliana PIRIN* genes have distinct activities in various tissue types throughout the development of the Arabidopsis plant. Histochemical staining of tissues from plants expressing β‐glucuronidase (GUS) fused to each of the *PIRIN* (*PRN*) promoters. Promoter activity of *PRN1* (a–f), *PRN2* (g–l), *PRN3* (m–r), *PRN4* (s–x) in the cotyledon (a, g, m, s), the root tip (b, h, n, t), the maturation zone of 5‐d‐old roots (c, i, o, u), the vascular bundle of inflorescence stems in 6‐wk‐old plants (d, j, p, v), and the hypocotyls of 8‐wk‐old plants (e, f, k, l, q, r, w, x). (f, l, r, x) are magnified images from (e, k, q, w), respectively. Bars, 100 μm. Arrowheads and arrows indicate examples of GUS activity in xylem parenchyma cells adjacent to xylem vessels and in xylem vessels, respectively.

First of all, we analysed promoter activities for the *PRN* genes by histochemical β‐glucuronidase (GUS) assays in transgenic lines carrying each of the *PRN* promoters coupled with GUS. GUS staining revealed that the promoter of *PRN1* is mainly active at the seedling stage and in epidermal cells of inflorescence stems, but not in the vasculature of the stem or the hypocotyl of mature plants (Fig. 1a–f). In contrast, the promoters of *PRN2*, *PRN3* and *PRN4* are active in vascular tissues at both seedling and mature plant stages (Fig. 1g–x). The *PRN3* promoter is active both in the phloem and xylem tissues of the stem and the hypocotyl (Fig. 1p–r). Notably, the *PRN2* promoter activity is restricted to xylem cells next to the vessel elements (Fig. 1j–l), while the promoter for *PRN4* is active in both developing xylem vessels and their neighbouring cells (Fig. 1v–x). Also, promoter activity of the *PRN2* homologue in *Populus* (*PttPRN2*; Potri.002G231900) is localised specifically to ray cells neighbouring the vessel elements in transgenic hybrid aspen (*Populus tremula *×* tremuloides*) trees carrying a pro*PttPRN2::GUS* construct (Fig. S2).

**Figure 2:**
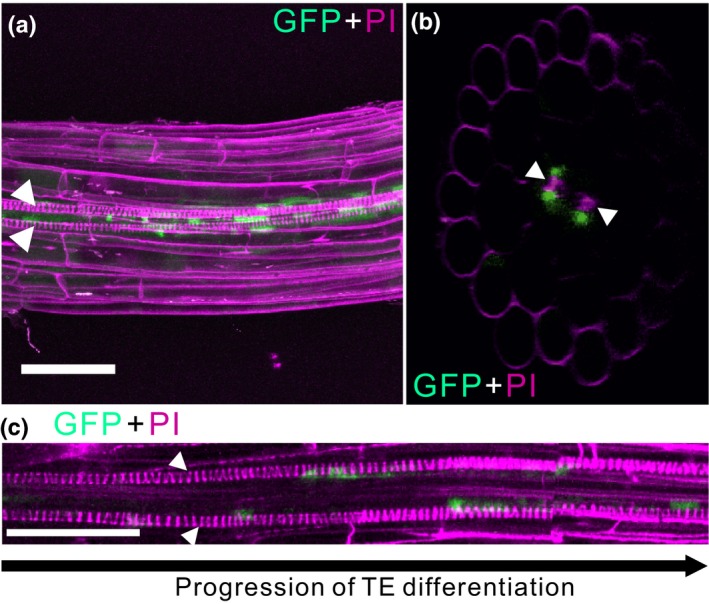
PRN2 is localised to cells adjacent to vessel elements in Arabidopsis vasculature. Confocal laser scanning microscopy (CLSM) imaging of pro*PRN2::PRN2:GFP* seedlings. (a) Maximum intensity projection of the main root of a 4‐d‐old seedling stained with propidium iodide (PI). (b) Optically reconstructed transverse section from (a). (c) Maximum intensity projection of CLSM imaging of the main root’s vasculature in a 4‐d‐old seedling similar to (a), with the leftmost vessel elements being the first ones with visible secondary cell walls (SCWs) in the two protoxylem files. White arrowheads indicate protoxylem vessels. Bars, 50 µm.

The activity of the *PRN2* promoter specifically in xylem elements neighbouring the vessels supports the function of PRN2 in noncell‐autonomous lignification of the vessel elements. We next analysed the localisation of the PRN2 protein in the roots of 4‐d‐old seedlings carrying a pro*PRN2::PRN2:GFP* construct. Confocal laser scanning microscopy (CLSM) revealed localisation of the PRN2:GFP fusion protein in cells next to the vessel elements but no signal in the vessel elements themselves (Fig. 2). The PRN2:GFP fusion protein appeared in this location prior to the death of the vessel elements (Fig. S3). Altogether, both the *PRN2* promoter activity and the PRN2 protein localisation support the noncell‐autonomous function of PRN2 in vessel lignification.

### PRN2 acts as a negative regulator of S‐type lignin accumulation

**Figure 3:**
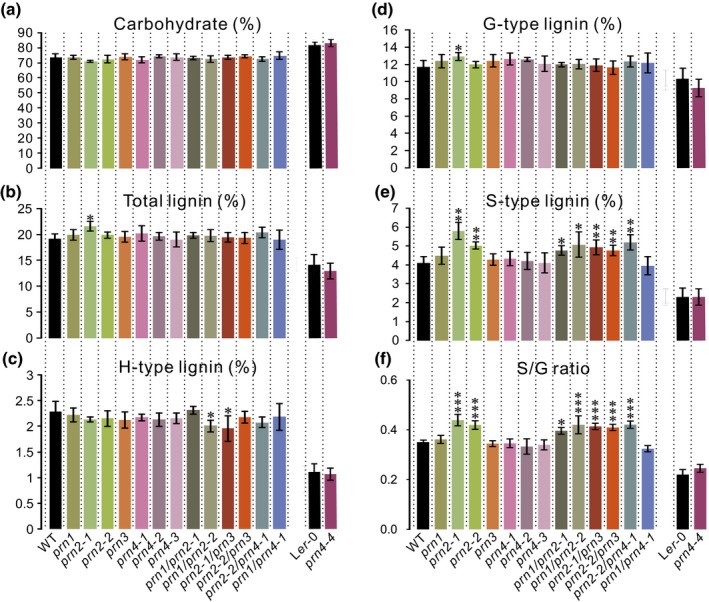
The effect of the different Arabidopsis PRN proteins on the secondary cell wall composition in hypocotyls. Relative content (percentage of detected cell wall components) of carbohydrates (cellulose and hemicelluloses) (a), total lignin (b), H‐type lignin (c), G‐type lignin (d) and S‐type lignin (e), and the S‐type : G‐type lignin ratio (f) in the cell walls of 8‐wk‐old wild‐type (WT) and single and double mutants for the *PRN* genes, analysed by pyrolysis–gas chromatography/mass spectrometry. All mutants are in Col‐0 background, except for *prn4‐4* which is in L*er*‐0 background. Error bars indicate ± SD. For each genotype, five biological replicates were analysed, each composed of a pool of three hypocotyls. The asterisks indicate statistically significant differences from the corresponding WT background, tested by Welch’s *t*‐test (*, *P* < 0.05; **, *P* < 0.01; ***, *P* < 0.001).

**Figure 4:**
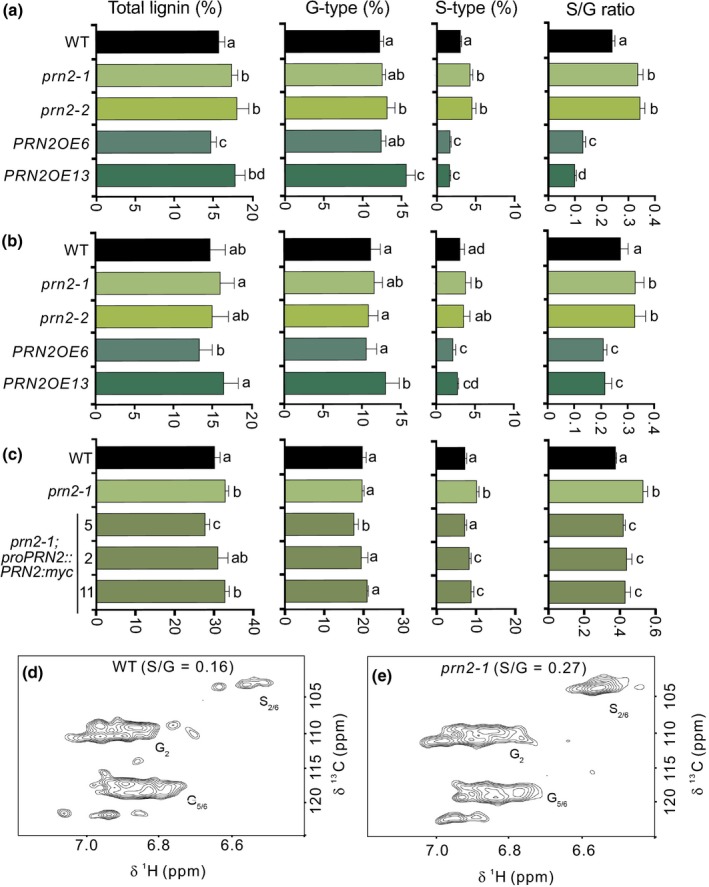
PRN2 affects lignin composition in the secondary xylem tissues of stems and hypocotyls. (a–c) Pyrolysis–gas chromatography/mass spectrometry analysis of hypocotyls (a, c) and stems (b) from 8‐wk‐old soil‐grown Arabidopsis plants. Relative content (percentage of detected cell wall components) of total lignin, G‐type lignin and S‐type lignin, and the S‐type : G‐type lignin ratio is shown for *prn2* mutants, *PRN2*‐overexpressor lines *PRN2OE6* and *PRN2OE13*, and *prn2* complementation lines (*prn2‐1* pro*PRN2::PRN2:myc*). For each genotype, five biological replicates were analysed, each composed of a pool of three hypocotyls (a, c) or stems (b). Lines that do not share any letter are significantly different (*P* < 0.05) from each other according to post ANOVA Fisher’s test. Error bars indicate ± SD. The experiment was repeated at least three times with similar results. (d, e) The aromatic region of the 2D HSQC NMR spectra of wild‐type (WT) (d) and *prn2‐1* (e). Two hundred bark‐peeled hypocotyls from each of WT and the *prn2‐1* mutant were harvested and pooled to obtain adequate lignin extracts for the analysis. Peaks from the S‐type and G‐type lignin are indicated by ʻSʼ and ‘G’, respectively and the numbers following these letters correspond to the positions in the aromatic rings that give rise to these peaks.

To investigate if the Arabidopsis PRN proteins function in xylem lignification, hypocotyl material from 8‐wk‐old T‐DNA insertion lines (Fig. S4) was analysed for each of the four *PRN* genes using pyrolysis–gas chromatography/mass spectrometry (Py‐GC/MS). Py‐GC/MS yields pyrolytic products that can be used to quantify the relative content of carbohydrates and the three different types (G‐, S‐ and H‐types) of lignin based on their respective MS peak area as a proportion of the cumulative area from all peaks (Gerber *et al*., 2012). In this analysis, 127 pyrolytic degradation products were analysed in 14 mutants, including single mutants *prn1, prn2‐1, prn2‐2, prn3, prn4‐1, prn4‐2, prn4‐3* and *prn4‐4*, as well as double mutants *prn1 prn2‐1, prn1 prn2‐2, prn2‐1 prn3, prn2‐2 prn3, prn2‐2 prn4‐1* and *prn1 prn4‐1,* and compared with the corresponding wild‐type plants (Fig. 3). Interestingly, the two *prn2* single mutants and the double mutants derived from crosses with the *prn2* mutants showed significantly higher relative content of S‐type lignin when compared with the Col‐0 wild‐type (Fig. 3e), supporting a function for PRN2 in lignification. This function was validated by analysis of the *prn2* mutants together with the *PRN2‐*overexpressing transgenic lines (*PRN2OE 6,13,29),* carrying a *35S::PRN2* construct, in both hypocotyl and stem material. Consistent with the increased relative content of S‐type lignin in the *prn2* mutants, Py‐GC/MS analysis showed lower relative content of S‐type lignin in the *PRN2‐*overexpressing lines, compared with the wild‐type (Figs 4a,b, S5). Py‐GC/MS analysis on hypocotyls of three PRN2 complementation lines (pro*PRN2::PRN2:myc* in *prn2‐1* background) showed partial complementation of the S‐type lignin content (Fig. 4c). Furthermore, two‐dimensional nuclear magnetic resonance spectroscopy (2D‐NMR) on dissolved hypocotyl cell wall material (Lu & Ralph, 2003) revealed an increased S : G ratio in the *prn2‐1* mutant (S : G = 0.27) compared with wild‐type (S : G = 0.16) (Fig. 4d,e). While the increase in the S‐type lignin of *prn2* was always consistent, increased relative content of G‐type lignin as well as increased total lignin content was somewhat variably found in *prn2* mutants compared with the wild‐type (Figs 3, 4a,b). Transgenic hybrid aspen trees with up to 40% reduction in the expression of *PttPRN2* showed also a slight tendency towards higher lignin S : G ratios (Fig. S2d–f). Taken together, the results support the action of PRN2 in suppressing S‐type lignin accumulation.

The effect of PRN2 on lignin accumulation was corroborated by comparative phenolic profiling of the *prn2* stem material using UHPLC–MS, which enables detection of several classes of aromatic compounds including benzenoids, phenylpropanoids, flavonols and oligolignols (Vanholme *et al*., 2012). In total, 6414 peaks were detected. The results showed 28 and 13 peaks with a significantly higher intensity in the *prn2‐1* and *prn2‐2* mutants compared with the wild‐type, respectively, of which nine were common to both alleles. No compounds were found with a significantly lower abundance in both. The nine peaks with significantly higher intensity appeared to be derived from eight compounds. Based on MS/MS fragmentation, seven of these (compounds 1–7) could be characterised as oligolignols (Table 1), containing at least one G unit and one S unit. The structure of the two other compounds could not be determined.

**Table 1:** Phenolic profiling of *prn2* and wild‐type (WT) stems.

	*m/z*	RT (min)	Name	Wild‐type	*prn2‐1*		*prn2‐2*	
				Average ± SD	Average ± SD	Ratio	Average ± SD	Ratio
1	583.218	14.71	G(8‐O‐4)S(8‐5)G 1	5485 ± 2151	13146 ± 4007	2.4	12786 ± 3073	2.3
2	583.218	16.93	G(8‐O‐4)S/G(8‐8)G/S 1*	377 ± 406	2521 ± 1140	6.7	2270 ± 717	6.0
3	583.217	17.75	G(8‐O‐4)S/G(8‐8)G/S 2*	9 ± 18	305 ± 249	36	184 ± 128	22
4	613.229	16.66	G(8‐O‐4)S(8‐8)S	499 ± 524	2886 ± 1303	5.8	2766 ± 722	5.5
5	809.305	17.97	G(8‐O‐4)S(8‐8)S(4‐O‐8)G 1	1529 ± 1395	8834 ± 3803	5.8	7847 ± 3141	5.1
6	809.305	18.67	G(8‐O‐4)S(8‐8)S(4‐O‐8)G 2	782 ± 780	5362 ± 2321	6.9	4553 ± 1983	5.8
7	839.316	17.72	G(8‐O‐4)S(8‐8)S(4‐O‐8)S	14 ± 29	313 ± 315	22	245 ± 186	17

All compounds differ between the *prn2* mutants and the WT with statistical significance (for detailed description, see the Materials and Methods section). ʻRatioʼ stands for average peak intensity in each of the *prn2* mutants divided by the average peak intensity of the WT. SD, standard deviation; RT, retention time. Nine biological replicates of Arabidopsis were analysed in each genotype. The asterisks indicate that the exact identity of these compounds could not be determined but that they were either G(8‐O‐4)S(8‐8)G or G(8‐O‐4)G(8‐8)S isomers.

### PRN2 controls lignification in a noncell‐autonomous manner

**Figure 5:**
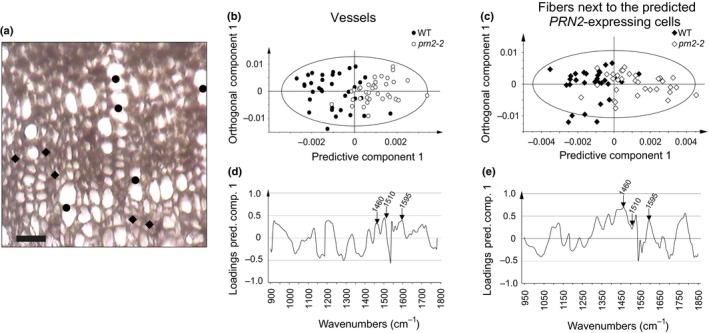
PRN2 acts in a noncell‐autonomous manner on xylem lignification in Arabidopsis. (a) White‐light image of a transverse section showing example positions of the extracted representative FT‐IR spectra; circles indicate cell walls between two vessel elements, and diamonds indicate cell walls between two xylem fibres that are located next to the predicted *PRN2*‐expressing cells. The *PRN2*‐expressing cells were predicted on the basis of the results from the pro*PRN2::GUS* plants (see Fig. 1k,l). The image was taken at ×15 magnification (used by the Cassegrain objective of the FT‐IR microscope). Bar, 25 µm. (b–e) FT‐IR microspectroscopic analysis of vessel elements (b, d) and fibres next to the predicted *PRN2*‐expressing fibres (c, e), in the secondary xylem of the hypocotyls of *prn2‐2* mutant and wild‐type (WT). Spectra were collected from three 8‐wk‐old plants per genotype, with at least 10 spectra per plant. Models always use 1 + 2 (predictive + orthogonal) components. (b, c) OPLS‐DA scores plots showing the separation between *prn2‐2* (white symbols) and WT (black symbols) in vessel–vessel walls (b) and cell walls between two fibres located next to the predicted *PRN2*‐expressing cells (c). Each symbol represents one measurement. Model details: vessel elements: *N* = 60, R2X(cum) = 0.56, R2Y(cum) = 0.39, Q2(cum) = 0.165; fibres next to the predicted *PRN2*‐expressing fibres: *N* = 64, R2X(cum) = 0.803, R2Y(cum) = 0.466, Q2(cum) = 0.310. Q2(cum) values stand for the predictive ability of the model, with higher values (closer to the maximum 1) generally meaning better separation for the same dataset. (d, e) The corresponding correlation‐scaled loadings plots for the predictive components, showing factors separating WT from *prn2‐2* in vessel elements (d) and fibres next to the predicted *PRN2*‐expressing fibres (e). Bands on the negative side of the plots have higher relative intensity in the spectra of wild‐type plants, whereas bands on the positive side have higher relative intensity in the spectra of *prn2‐2*. Loadings higher than 0.4 (more than 40% correlation) can be considered indicative of changes that are specific to their respective genotypes. The bands corresponding to the aromatic –C=C– skeletal vibrations at 1510 and 1595 cm^−1^, indicating prominent bands that can be correlated to lignin, and a band around 1460 cm^−1^ are indicated by arrows. The experiments were repeated twice with similar results.

Next, we investigated whether the apparent localisation of the PRN2 protein next to the vessel elements affects cell wall chemistry of the neighbouring vessels and fibres. Transverse sections from the hypocotyls were analysed using Raman microspectroscopy, which enables chemotyping of individual cell walls with a micrometre lateral resolution (Gorzsás, 2017). PCA revealed that the cell wall chemotypes of both the vessels (measured from cell walls between two vessels) and the fibres (measured from cell walls between two fibres) were different between the *prn2‐1* mutant and the wild‐type (Fig. S6). The cell walls of the *PRN2* complementation line 5 carrying pro*PRN2::PRN2:myc* in the *prn2‐1* background were more similar to the wild‐type than to the *prn2‐1* mutant (Fig. S6). The *prn2‐1* mutant exhibited increased fluorescence during Raman microspectroscopic measurements in both vessels and fibres to a level that disabled identification of compounds contributing to the separation between the genotypes. Nevertheless, this was indicative of lignin‐related changes in the cell wall matrix of *prn2‐1*, as lignin is primarily responsible for the fluorescence in these samples. We therefore complemented Raman microspectroscopy with the analysis of the cell walls in transverse section of hypocotyls of the wild‐type and *prn2‐2* mutant by FT‐IR microspectroscopy combined with OPLS‐DA. This method generally allows robust and sensitive chemotyping of individual cells (Gorzsás *et al*., 2011), but has lower (diffraction limited) spatial resolution than Raman microspectroscopy. Cell‐specific FT‐IR spectra were collected from cell walls of vessels and fibres located next to the predicted *PRN2*‐expressing cells (Fig. 5a). Pairwise OPLS‐DA comparisons of the FT‐IR spectra showed separation between *prn2‐2* and wild‐type in both cell types (Fig. 5b,c). Lignin‐related differences could be tracked in the corresponding correlation‐scaled loadings plots (Fig. 5d,e) via the band intensities of the aromatic –C=C– skeletal vibrations at 1595 and 1510 cm^−1^ (Faix, 1991). The intensity of the band at 1595 cm^−1^ (often correlated with S‐type lignin) was increased in *prn2‐2* in comparison with the wild‐type in both the vessels (Fig. 5b,d) and in the fibres next to the predicted *PRN2*‐expressing cells (Fig. 5c,e), while the band at 1510 cm^−1^ (indicative of more cross‐linked structures and often correlated with G‐type lignin) was increased in *prn2‐2* in comparison with the wild‐type only in the vessels (Fig. 5b,d). The intensity of an additional band at *c*. 1460 cm^−1^, which indicates changes in =CH and/or –C–H vibrations originating from either lignin or noncellulosic polysaccharides (Olsson *et al*., 2011), was also increased in *prn2‐2* (Fig. 5d,e). These data support a function of PRN2 in suppressing especially the S‐type lignin accumulation in a noncell‐autonomous manner.

Transverse sections of inflorescence stems were chemotyped by Raman microspectroscopy (Fig. S7). The cell wall chemistry of the interfascicular fibres did not differ between *prn2‐2* and wild‐type. In contrast, increased intensities were observed in both the vessel elements and the fibres of the vascular bundles in *prn2‐2* compared with the wild‐type for the typical lignin‐related bands at 1595 and 1660 cm^−1^. These increases and the slight shift in the band at 1595 cm^−1^ suggest structural or compositional changes in addition to an increase in lignin content in *prn2‐2*. Thus, PRN2 modulates lignin deposition in the vascular bundles of the inflorescence stems in accordance with its expression pattern in these tissues.

### PRN2 influences the expression of the lignin‐biosynthetic genes

**Figure 6:**
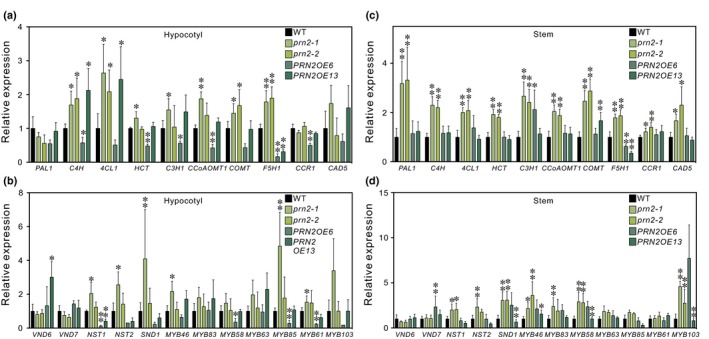
PRN2 suppresses the expression of lignin‐biosynthetic genes and some of the transcription factors regulating secondary cell wall biosynthesis in Arabidopsis. Expression of lignin‐biosynthetic genes (a, c) and transcription factors related to secondary cell wall biosynthesis (b, d) in the hypocotyls (a, b) and the stems (c, d) of *prn2‐1, prn2‐2* and the *PRN2‐*overexpressor lines *PRN2OE6* and *PRN2OE13*. The expression levels were normalised to the expression in the wild‐type (WT). For each genotype, five biological replicates were analysed, each composed of a pool of three hypocotyls (a, b) or stems (c, d). *PDF2* and *UBQ10* were used as reference genes. Error bars indicate ± SD. The asterisks indicate statistically significant difference from the WT by Welch’s *t*‐test (*, *P* < 0.05; **, *P* < 0.01).

PRN protein functions in mammals as a transcriptional co‐regulator. We have earlier demonstrated that PRN2 is localised in the cytoplasm and in the nucleus (Zhang *et al*., 2014), supporting possible function in transcriptional control. We therefore tested whether PRN2 function on S‐type lignin accumulation could involve transcriptional control of the lignin‐biosynthetic genes. The expression of genes encoding lignin‐biosynthetic enzymes as well as secondary cell wall biosynthesis‐related transcription factors were analysed in the hypocotyls and stems of the *prn2* mutants and the *PRN2*‐overexpressor lines. Increased expression was apparent in *prn2* mutants for several lignin‐biosynthetic genes, including *C4H* (cinnamate 4‐hydroxylase), *4CL1* (4‐coumarate:CoA ligase 1), *C3H1* (*p*‐coumarate 3‐hydroxylase 1), *CCoAOMT1* (caffeoyl‐CoA *O*‐methyltransferase 1), *COMT* (caffeic acid *O*‐methyltransferase) and *F5H1* (ferulate 5‐hydroxylase 1) compared with the wild‐type (Fig. 6a,c). It was interesting to note that the most consistent gene expression changes (increased expression in *prn2* mutants and lower expression in *PRN2* overexpressors in both tissue types) were present for *F5H1* which is specific to S‐type lignin biosynthesis. Almost all studied transcription factors except for VND6 and VND7 showed either a trend or statistically significant increase in expression in *prn2* compared with wild‐type especially in the stems, while suppressed expression was evident for several of the transcription factor genes in the *PRN2*‐overexpressor lines (Fig. 6b,d). Taken together, the results support PRN2 function in suppressing the expression of both the lignin‐biosynthetic genes and the secondary cell wall regulatory transcription factors.

## Discussion

Lignin deposition can occur in a noncell‐autonomous manner whereby lignification of xylem elements is assisted by cells neighbouring the lignifying cells (Hosokawa *et al*., 2001; Pesquet *et al*., 2013; Smith *et al*., 2013; De Meester *et al*., 2018). The underlying mechanism has, however, remained unknown. Here we identify PRN2 as a factor modulating noncell‐autonomous lignification in Arabidopsis. *PRN2* was shown to be strictly expressed in the xylem elements next to the lignifying vessels, and from within these *PRN2‐*expressing cells, to suppress accumulation of lignin in the neighbouring cells not expressing *PRN2*. These results imply that plants have the potential to increase lignin content of xylem elements by boosting noncell‐autonomous lignification even *post mortem,* that is after the death of the xylem elements. Noncell‐autonomous lignification provides, therefore, yet another level of control as well as plasticity to modify lignin deposition of xylem elements in response to changing developmental or environmental cues.

### PRN2 marks the xylem cells contributing to noncell‐autonomous lignification

In earlier studies, Smith *et al*. (2013, 2017) defined in Arabidopsis stem tissues the cell types that contribute to noncell‐autonomous lignification. Modification of lignin biosynthesis in specific cell types revealed that parenchymatic, nonlignifying cells as well as xylary fibres provide lignin precursors to neighbouring vessel elements and fibres in a noncell‐autonomous fashion. Lignification of the interfascicular fibres, conversely, seemed mostly cell autonomous. Furthermore, Smith *et al*. (2017) showed that the contribution of each cell type depends on the tissue composition of the vascular bundles. For instance, in the young part of the stem, where no fibres were yet present, only the parenchymatic cells contributed to noncell‐autonomous lignification, while in the older parts of the stem both parenchymatic cells and fibres contributed. Our results on the *PRN2* promoter activity in the parenchymatic cells and xylary fibres of the vascular bundles but not in the neighbourhood of the interfascicular fibres in the lower part of the stem (Fig. 1j–l) is consistent with these results, indicating that PRN2 might be a good marker for the cells contributing to noncell‐autonomous lignification. Accordingly, PRN2 influenced lignification of xylem elements in the vascular bundles but not of the interfascicular fibres (Fig. S7). Noncell‐autonomous lignification has not been studied earlier during secondary growth of the hypocotyl. We could show in the secondary xylem of the hypocotyl that *PRN2* promoter activity was specific to the vessel‐neighbouring fibres that had thick secondary cell walls and that did not in any way seem different from the rest of the secondary xylem fibres of the hypocotyl (Fig. 1k,l). Interestingly, in the secondary xylem tissues of *Populus* stem, the *Populus PRN2* promoter was active only in the ray cells next to the vessel elements (Fig. S2a–c). Expression of the lignin‐biosynthetic genes has also repeatedly been reported in the cells that are located next to the lignifying vessel elements of the secondary xylem (Hauffe *et al*., 1991; Feuillet *et al*., 1995; Chen *et al*., 2000; Baghdady *et al*., 2006). These results suggest that it is the vessel‐neighbouring ray cells that contribute to noncell‐autonomous lignification during secondary growth of the stem. Arabidopsis hypocotyls do not seem to have ray cells (Chaffey *et al*., 2002), and it is possible that, in Arabidopsis, the *PRN2*‐expressing xylem fibres have adopted some of the functions, including contribution to noncell‐autonomous lignification, that are normally performed by the ray cells in other species.

### PRN2 suppresses primarily the accumulation of S‐type lignin in the neighbourhood of the vessels

The preferred expression of *PRN2* next to the vessel elements suggests that noncell‐autonomous lignification is more important for the vessel elements than for the fibres. This seems plausible also due to the fact that, unlike the xylem fibres, the vessel elements differentiate and die very rapidly and might have opted for a strategy relying on noncell‐autonomous lignification due to their short lifetime (Courtois‐Moreau *et al*., 2009). It was striking that the PRN2 effect on lignin accumulation was strongest for the S‐type lignin, which together with the fact that the secondary walls of the vessel elements are enriched in G‐type lignin suggests that PRN2 function is primarily related to suppression of S‐type lignin of the vessel elements. PRN2 localisation in cells next to the vessel elements supports this hypothesis. From this situation, it follows that the enrichment of G‐type lignin in the vessel elements is at least partially acquired by suppression of S‐type lignin accumulation in a noncell‐autonomous, PRN2‐dependent manner. The mechanism seems, however, somewhat unrefined as excess lignin accumulation in the *prn2* mutants was not restricted to the vessel elements but was present also in fibres neighbouring the *PRN2*‐expressing cells (Figs 5, S7). In line with this, chemotyping of *Populus* woody tissues revealed that changes in lignin monomer composition between the different xylem cell types were gradual, the vessel‐neighbouring fibres displaying lignin composition that was intermediate between that of the vessel elements and the more distant fibres (Gorzsás *et al*., 2011).

Lignification is known to be regulated at three different levels: the biosynthesis of monolignols within the cell, their export through the plasma membrane into the cell wall, and polymerisation within the cell wall. To date, very little information is known about monolignol transport and how it could influence lignin composition. A transporter for *p*‐coumaryl alcohol is known (Alejandro *et al*., 2012), but transporters for coniferyl and sinapyl alcohol remain to be characterised. Lignin polymerisation in xylem is, according to current knowledge, influenced mainly by the availability of the monolignols (Tobimatsu & Schuetz, 2019). Hence, the most tightly regulated step of lignification seems to be on the level of regulation of monomer biosynthesis within the cell. Our results support the function of PRN2 on S‐type lignin accumulation at this level by regulating the expression of the lignin‐biosynthetic genes and, in particular, the S‐type specific *F5H1* (Fig. 6). The human PRN protein is known to function as a transcriptional co‐regulator (Dechend *et al*., 1999; Liu *et al*., 2013), supporting function of PRN2 in transcriptional regulation of xylem lignification of vascular plants.

### Harnessing noncell‐autonomous lignification to counteract feedstock recalcitrance

Reduction in lignin content is desired in several downstream applications of the lignocellulosic raw material such as biochemical conversion of the carbohydrates to sugar moieties by enzymatic hydrolysis (i.e. saccharification). Reduction in the overall level of lignin content, however, causes undesirable side effects such as growth penalties, most likely to be due to impaired water transport in vessels that tend to collapse due to low lignin content. Efforts to reduce lignin in cell types other than vessels have also not been fully satisfactory (Yang *et al*., 2013; Vargas *et al*., 2016). The localised effect of noncell‐autonomous lignification offers an interesting alternative to modify cell wall chemistry of specific xylem cell types. Maintaining noncell‐autonomous lignification and hence lignification of the vessel elements in plants that have fibres with a low lignin content is a desired property that is expected to reduce recalcitrance of the lignocellulose to enzymatic hydrolysis without necessarily compromising the growth of the plants. A few recent reports have given support to this approach. Smith *et al*. (2013) reported increased saccharification without growth penalty in Arabidopsis stems in which lignin biosynthesis was disrupted by an artificial microRNA construct for *CCR1* under the control of the *CesA7* promoter that reduced lignin biosynthesis in all lignifying cells but allowed lignification of the vessels through noncell‐autonomous lignification by the nonlignifying, parenchymatic cells. Similarly, significant increases in saccharification without a growth penalty were obtained in Arabidopsis stems where lignification was reintroduced to the vessel elements of *ccr1* mutant by transgenic expression of *CCR1* under the control of a synthetic promoter containing the vessel‐specific SNBE domains (De Meester *et al*., 2018). In both cases, it is likely that the increases in saccharification were gained because of low lignification in the interfascicular fibres that constitute the main lignocellulosic mass of Arabidopsis stem. Interfascicular fibres are not the dominating lignified cell type in major lignocellulosic feedstocks such as forest trees, and it will be interesting to study whether noncell‐autonomous lignification could be harnessed also in trees to improve the biochemical conversion properties of lignocellulose. Our results on PRN2 provide a new tool to investigate this aspect. Reintroduction of lignification into the *PRN2*‐expressing cells in trees that are hampered in lignin biosynthesis might result in low lignin lignocellulosic biomass without affecting the biomass yield.

## Author contributions

HTuominen and WB conceived the study, BZ, SE, MH, YW, AG, BS, HTurumtay, HTuominen and RV performed the experiments, BZ, SE, RV and HTuominen analysed the data, BZ, SE and HTuominen wrote the manuscript with contributions from all the coauthors. [Correction added after online publication 11 November 2019: the contribution of author H Turumtay has been inserted.] BZ and BS contributed equally to this work.

## Competing interests

HTuominen is a member of the holding company Woodheads AB, a part‐owner of SweTree Technologies, which played no part in this work.

## Supporting information


**Fig. S1** Phylogenetic analysis of PRN proteins in plants.
**Fig. S2** Characterisation of the PRN2 function in hybrid aspen.
**Fig. S3** The expression of PRN2 is initiated during the lifetime of the vessel elements in Arabidopsis roots.
**Fig. S4** Characterisation of T‐DNA mutants for *PRN1*, *PRN3* and *PRN4* and a novel *PRN2* overexpression line.
**Fig. S5** PRN2 affects lignin composition in the secondary xylem tissues of stems.
**Fig. S6** Raman microspectroscopic analysis of vessel elements and xylem fibres in the secondary xylem of the hypocotyls.
**Fig. S7** Raman microspectroscopic analysis of interfascicular fibres, vascular bundle fibres and vessel elements in the secondary xylem of the stems.
**Table S1** All primer sequences used in this study.
Please note: Wiley Blackwell are not responsible for the content or functionality of any Supporting Information supplied by the authors. Any queries (other than missing material) should be directed to the *New Phytologist* Central Office.
