# Supplementary material for: PIRIN2 suppresses S‐type lignin accumulation in a noncell‐autonomous manner in Arabidopsis xylem elements
Source: New Phytol. 2019 Nov 11;225(5):1923–35. doi: 10.1111/nph.16271 (PMC7027918; doi:10.1111/nph.16271)
Supplement: Supplementary file 1 — Fig. S1 Phylogenetic analysis of PRN proteins in plants. Fig. S2 Characterisation of the PRN2 function in hybrid aspen. Fig. S3 The expression of PRN2 is initiated during the lifetime of the vessel elements in Arabidopsis roots. Fig. S4 Characterisation of T‐DNA mutants for PRN1, PRN3 and PRN4 and a novel PRN2 overexpression line. Fig. S5 PRN2 affects lignin composition in the secondary xylem tissues of stems. Fig. S6 Raman microspectroscopic analysis of vessel elements and xylem fibres in the secondary xylem of the hypocotyls. Fig. S7 Raman microspectroscopic analysis of interfascicular fibres, vascular bundle fibres and vessel elements in the secondary xylem of the stems. Table S1 All primer sequences used in this study. Please note: Wiley Blackwell are not responsible for the content or functionality of any Supporting Information supplied by the authors. Any queries (other than missing material) should be directed to the New Phytologist Central Office. [file NPH-225-1923-s001.pdf]

New Phytologist Supporting Information

'Correction made after online publication 11 November 2019; Halbay Turumtay has been added to the author group.

Article title: **PIRIN2 suppresses S-type lignin accumulation in a non-cell-autonomous manner in Arabidopsis xylem elements**

Authors: Bo Zhang, Bernadette Sztojka, Sacha Escamez, Ruben Vanholme, Mattias Hedenström, Yin Wang, Halbay Turumtay, András Gorzsás, Wout Boerjan, and Hannele Tuominen

Accepted: 10 October 2019

The following Supporting Information is available for this article:

Figure S1. Phylogenetic analyses of PRN proteins in plants.

Figure S2. Characterization of the PRN2 function in hybrid aspen.

Figure S3. The expression of PRN2 is initiated during the lifetime of the vessel elements in Arabidopsis roots.

Figure S4. Characterisation of T-DNA mutants for *PRN1*, *PRN3* and *PRN4* and a novel *PRN2* overexpression line.

Figure S5. PRN2 affects lignin composition in the secondary xylem tissues of stems.

Figure S6. Raman microspectroscopic analysis of vessel elements and xylem fibers in the secondary xylem of the hypocotyls.

Figure S7. Raman microspectroscopic analysis of interfascicular fibers, vascular bundle fibers and vessel elements in the secondary xylem of the stems.

Table S1. All primer sequences used in this study.

Supplemental Figure 1

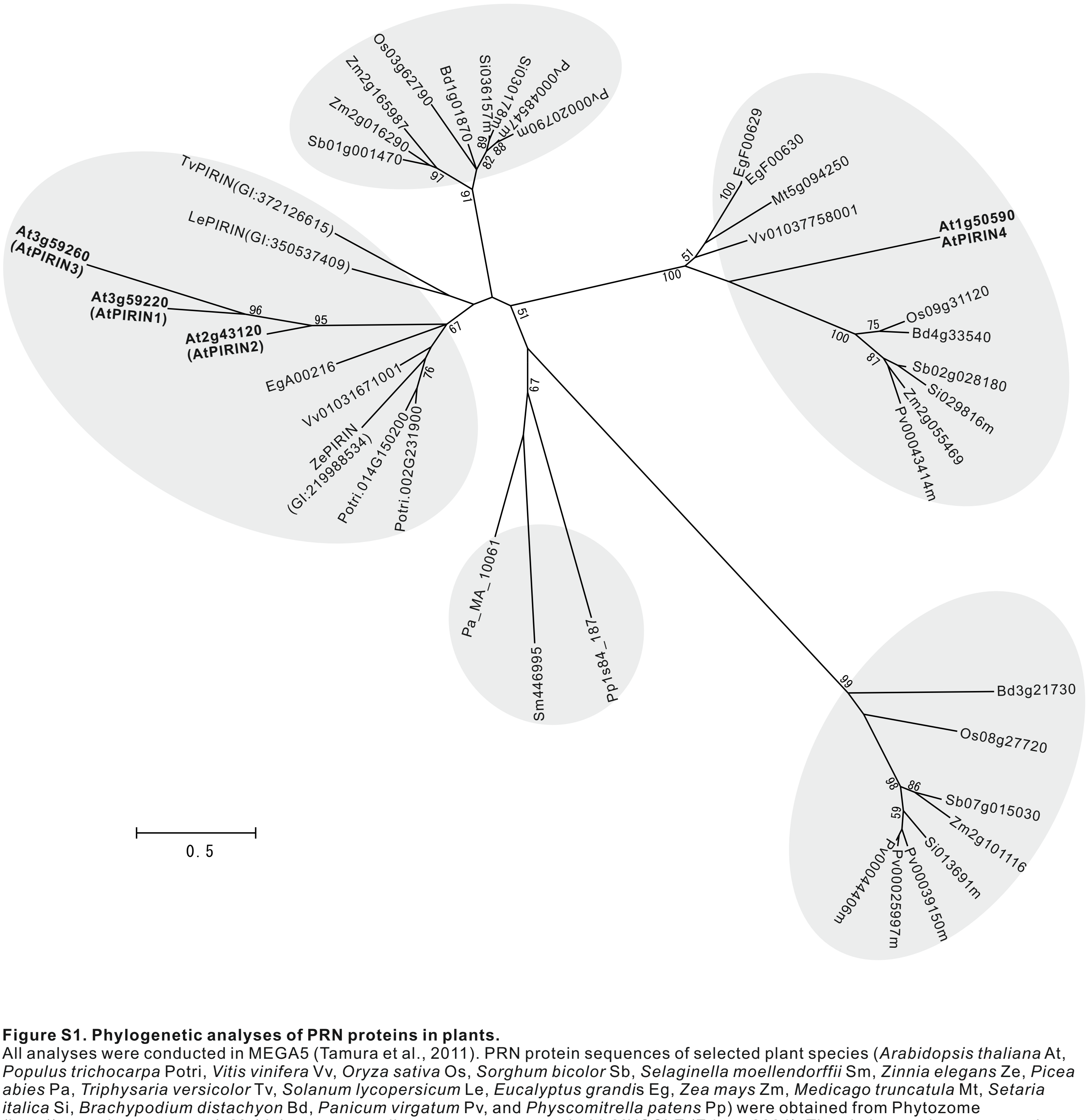

Figure S1. Phylogenetic analyses of PRN proteins in plants.
All analyses were conducted in MEGA5 (Tamura et al., 2011). PRN protein sequences of selected plant species (*Arabidopsis thaliana* At, *Populus trichocarpa* Potri, *Vitis vinifera* Vv, *Oryza sativa* Os, *Sorghum bicolor* Sb, *Selaginella moellendorffii* Sm, *Zinnia elegans* Ze, *Picea abies* Pa, *Triphysaria versicolor* Tv, *Solanum lycopersicum* Le, *Eucalyptus grandis* Eg, *Zea mays* Zm, *Medicago truncatula* Mt, *Setaria italica* Si, *Brachypodium distachyon* Bd, *Panicum virgatum* Pv, and *Physcomitrella patens* Pp) were obtained from Phytozome (http://www.phytozome.net). Multiple sequence alignments were created with MUSCLE (Edgar, 2004). The phylogenetic reconstruction was inferred by using the Maximum Likelihood method based on the Jones-Taylor-Thorn substitution model with gamma rate distribution (Guindon et al., 2010). The numbers at the nodes indicate bootstrap support calculated by RAxML bootstrapping using 1000 replications. The scale bar represents 0.5 substitutions per nucleotide position.

Supplemental Figure 2

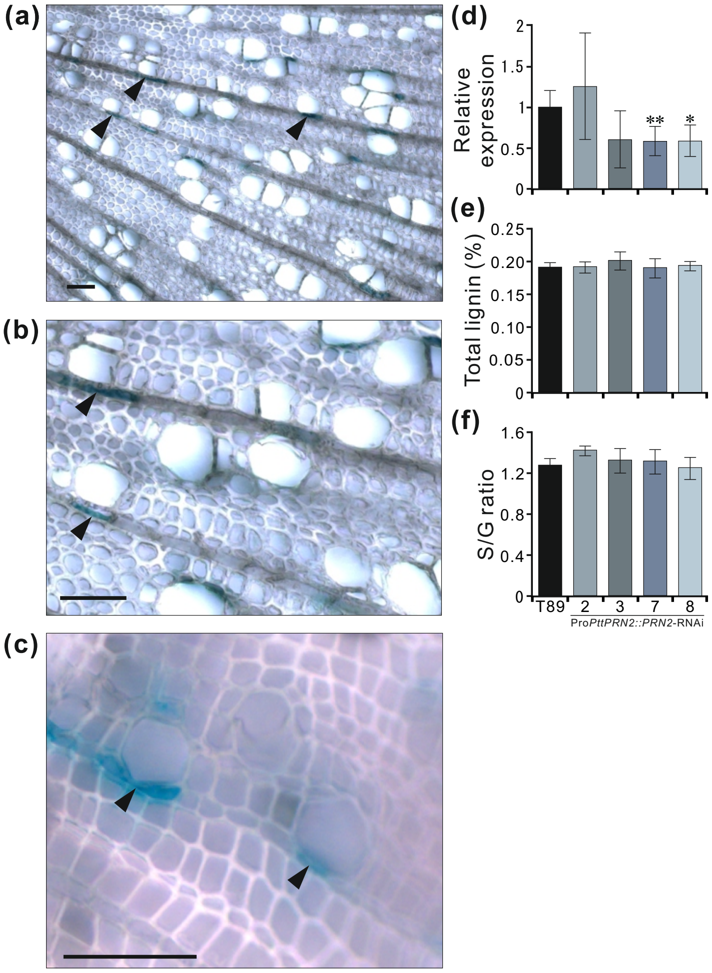

Figure S2. Characterization of the PRN2 function in hybrid aspen.
Histochemical GUS assay was performed for transverse sections of transgenic *Populus tremula x tremuloides* trees carrying a pro*PttPRN2::GUS* construct, grown for two months in the greenhouse (a, b) or for four weeks *in vitro* (c). Arrowheads indicate examples of GUS activity in cells adjacent to xylem vessels. The scale bars indicate 50 µm. (b) is a magnified version of (a).
(d) The expression level of the *PttPRN2* gene in *Populus tremula x tremuloides* trees expressing the Pro*PttPRN2::PRN2-RNAi* construct. Expression level is normalized to the T89 WT. Error bars indicate ±SD. The asterisks indicate statistically significant difference from the T89 WT by Welch's *t*-test (* p<0.05, ** p<0.01).
(e, f) Py-GC/MS analysis of the mature xylem from the stem of *Populus tremula x tremuloides* Pro*PttPRN2::PRN2-RNAi* lines, showing the relative content of total lignin (%) and the S/G-type lignin ratio. Error bars indicate ±SD.

Supplemental Figure 3

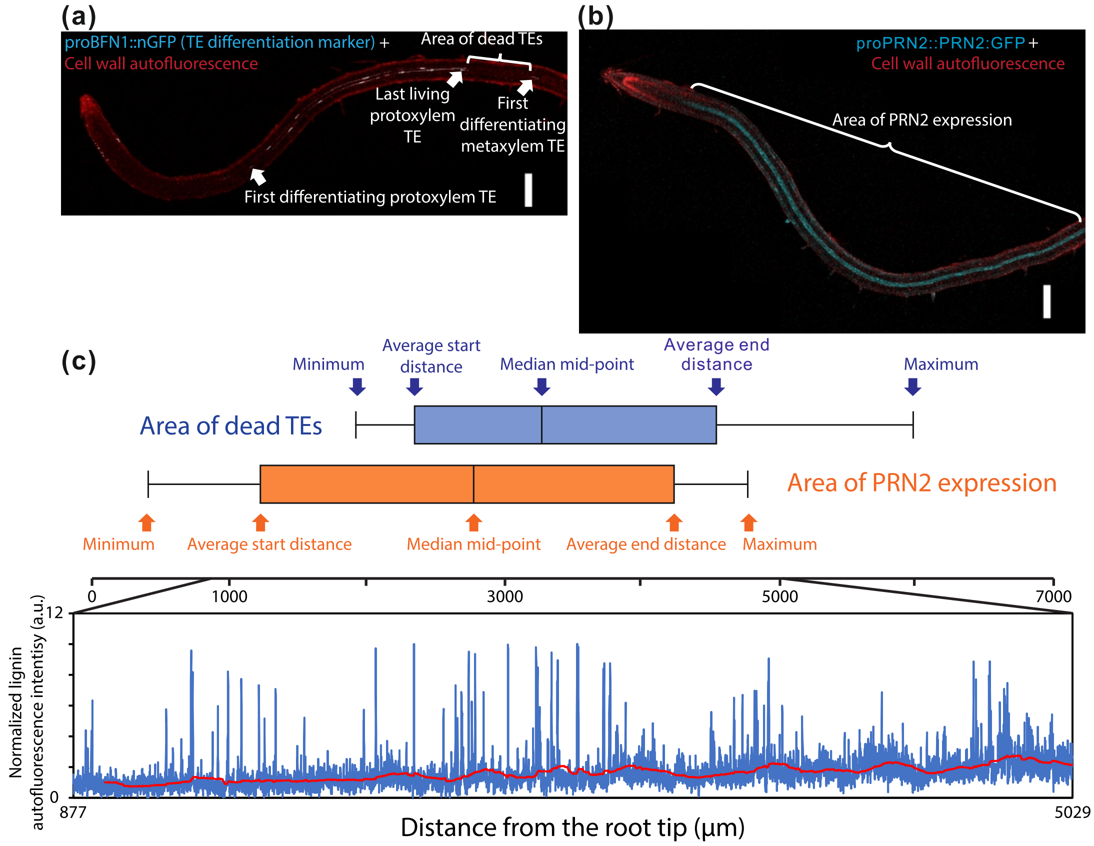

Figure S3. The expression of PRN2 is initiated during the lifetime of the vessel elements in Arabidopsis roots.
(a, b) Maximum intensity projections from confocal laser scanning microscopy observations of Arabidopsis seedlings' roots expressing the transcriptional marker for vessel cell death pro*BFN1::nGFP* (a) or pro*PRN2::PRN2:GFP* (b). In (a) the promoter of *BFN1*, a known marker for the differentiation of cell types whose differentiation terminates with cell death, drives the expression of a nuclear-targeted GFP (Bollhöner et al., 2013). The GFP signal closest to the root tip reveals when the latest formed protoxylem vessels start maturing. The loss of GFP indicates presence of dead protoxylem vessel elements, as indicated in the image. GFP signal appears again further from the root tip when metaxylem vessel elements start maturation.
(c) Localization (n = 8 biological replicates) of the area of 4-5 days old Arabidopsis seedlings' roots displaying dead protoxylem TEs but not yet any differentiating metaxylem TEs ("Area of dead TEs"; blue) and the PRN2 expression area (orange). The area of dead TEs and the PRN2 expression area overlap between ~2400 µm and ~4200 µm from the root tip, with a clear statistical overlap around 3000 µm. The lower panel shows lignin autofluorescence (blue) and its trend (moving averages, red) in a protoxylem cell file, showing a steady increase even at distances where TEs are likely dead.

Supplemental Figure 4

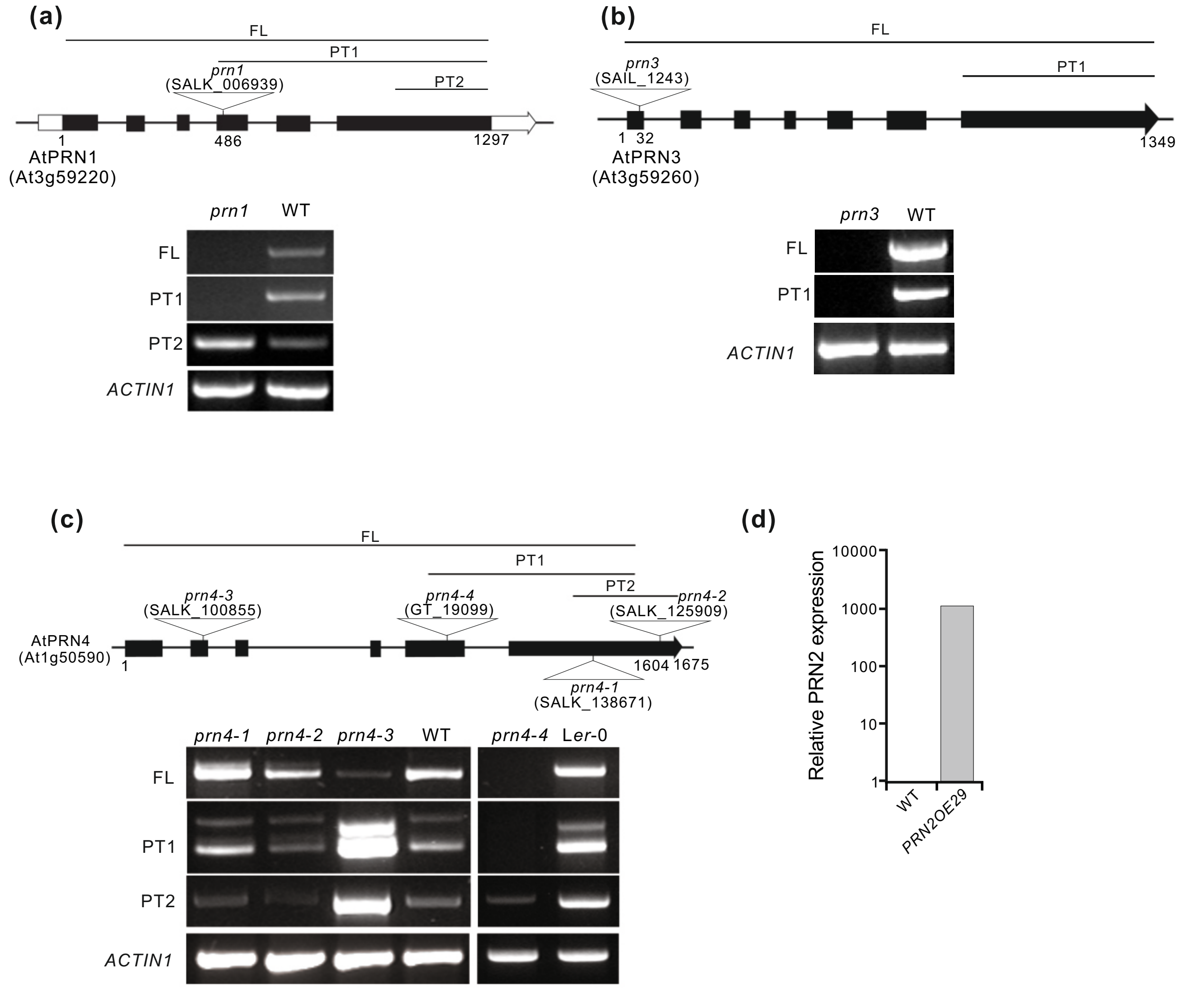

Figure S4. Characterisation of ArabidopsisT-DNA mutants for *PRN1*, *PRN3* and *PRN4* and a novel *PRN2* overexpression line.

Expression of *PRN1* (a), *PRN3* (b), and *PRN4* (c) is shown by RT-PCR using primers that resulted in amplification of either full length (FL) or partial transcript (PT1, PT2) as indicated on the top of each gene model. *ACTIN1* was used as reference gene. Exons are represented by filled black boxes, introns by lines, and T-DNA insertions by triangles. No full-length transcript of the corresponding *PRN* gene could be amplified by RT-PCR from any of the *prn1*, *prn3*, or *prn4-4* mutants, indicating that they are null mutants. Corresponding WT backgrounds (Col-0 and Ler-0) are included as controls. For PRN2, two null mutants *prn2-1* and *prn2-2* were described earlier in Zhang et al. (2014).

(d) The level of overexpression in the *PRN2*-overexpressing line 29 is shown by qRT-PCR. UBQ10 was used as reference

Supplemental Figure 5

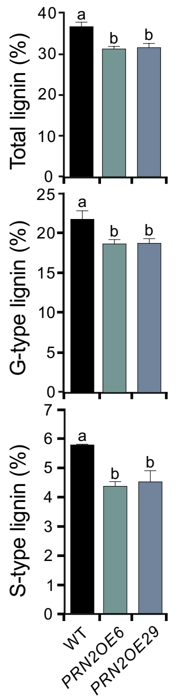

Figure S5. PRN2 affects lignin composition in the secondary xylem tissues of stems. Pyrolysis-gas chromatography/mass spectrometry analysis of stems from 8-week-old soil-grown Arabidopsis plants. Relative content (percentage of detected cell wall components) of total lignin, G-type lignin and S-type lignin is shown for the *PRN2*-overexpressor lines *PRN2OE6* and *PRN2OE*29. For each genotype, four biological replicates were analyzed, each composed of a pool of three stems. Lines that do not share any letter are significantly different ($p < 0.05$) from each other according to post ANOVA Fisher's test. Error bars indicate ±SD.

Supplemental Figure 6

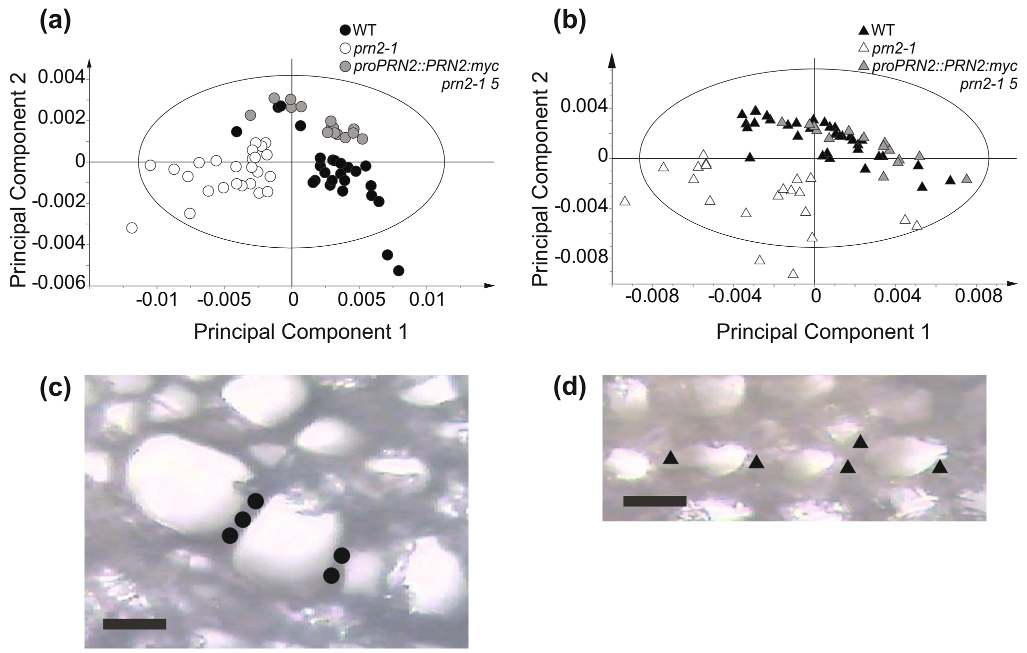

Figure S6. Raman microspectroscopic analysis of vessel elements and xylem fibers in the secondary xylem of the Arabidopsis hypocotyls.
PCA scores plots based on Raman microspectroscopic data, showing the separation between vessel elements (a; VE, circles) and xylem fibres (b; XF, triangles) of WT (black symbols), *prn2-1* (white symbols) and pro*PRN2::PRN2:myc prn2-1* line 5 (gray symbols). Spectra were specifically extracted from voxels representing pure vessel-vessel or fiber-fiber cell walls (see (c, d) for examples). Four 8-week-old plants were analyzed for each genotype. Each symbol represents one spectrum. Model details: vessel elements (a): 22 components, R2X(cum)=0.996, Q2(cum)=0.981, R2X [1] = 0.729, R2X [2] =0.0988, N=65; xylem fibers (b): 23 components, R2X(cum)=0.997, Q2(cum)=0.983, R2X [1] = 0.517, R2X [2] =0.35, N=71. (c, d) are white light images of transverse sections showing the positions of the extracted representative spectra. The images are taken at 50x magnification. The scale bars indicate 10 µm.

Supplemental Figure 7

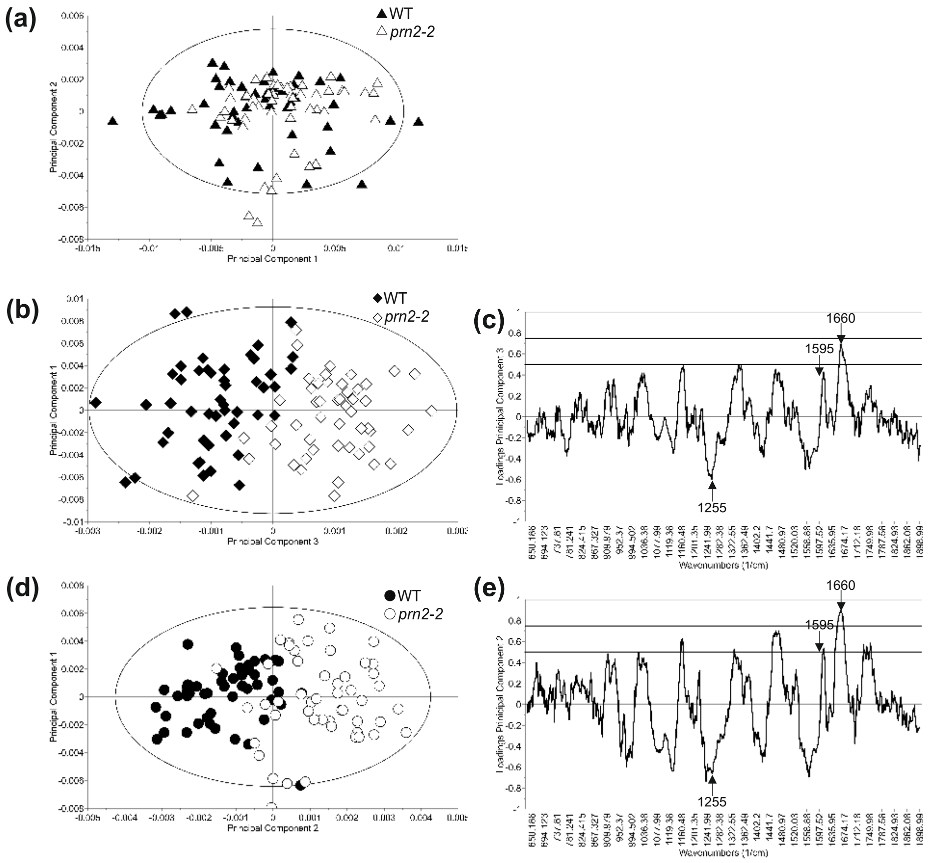

Figure S7. Raman microspectroscopic analysis of interfascicular fibers, vascular bundle fibers and vessel elements in the secondary xylem of Arabidopsis stems. PCA scores plots based on Raman microspectroscopic data, showing the separation between interfascicular fibers (a; triangles), vascular bundle fibers (b; diamonds) and vascular bundle vessel elements (d; circles) of WT (black symbols) and *prn2-2* (white symbols). Spectra were extracted from voxels representing pure vessel-vessel or fiber-fiber cell walls. Stems of five 8-week-old plants were analyzed for each genotype. Each symbol represents one spectrum. Model details: interfascicular fibers (a): 12 components, R2X(cum)=0.962, Q2(cum)=0.938, N=100; vascular bundle fibers. No genotypic differences were captured by any of the principal components or their combinations in the Raman data of interfascicular fibers, thus no loadings are shown. (b): 16 components, R2X(cum)=0.979, Q2(cum)=0.953 component 3: R2X=0.069, R2X(cum)=0.855, Q2=0,309, Q2(cum)=0.818, N=100; vascular bundle vessel elements (d): 27 components, R2X(cum)=0.987, Q2(cum)=0.958, component 2: R2X=0.198, R2X(cum)=0.65, Q2=0,341, Q2(cum)=0.629, N=100. (c, e) The corresponding correlation scaled loadings plot for the principal components that captured genotypic differences, showing factors separating WT from *prn2-2* in vascular bundle fibers and vessel elements, respectively. Bands on the negative side of the plots have higher relative intensity in the spectra of WT plants, whereas bands on the positive side have higher relative intensity in the spectra of *prn2-2* plants. Loadings higher than 0.4 (more than 40% correlation) can be considered indicative of changes that are specific to their respective genotypes. The bands corresponding to the aromatic -C=C- vibrations at 1595 and 1660 cm-1 are marked by arrows, indicating prominent bands that can be correlated to lignin (De Meester et al., 2018).

**Table S1. All primer sequences used in this study**		
**Sequence**	**Primer Name**	**Purpose**
AGAAGGAAGGTGAAGGAGCTG	SALK_006939.41.15.x-R	*prn1* T-DNA line genotyping
AGAAGGAAGGTGAAGGAGCTG	SALK_006939.38.30.x-R	*prn1* T-DNA line genotyping
TGTGACCTTTGAAGTCTTGGTG	SM_3_15394-L	*prn2-1* T-DNA line genotyping
CTCGTTTTTCTGTCATTTGACTG	SM_3_15394-R	*prn2-1* T-DNA line genotyping
CCGAATCAACAACAATGAAAAC	SALK_079571.50.85.x-L	*prn2-2* T-DNA line genotyping
TGTGACCTTTGAAGTCTTGGTG	SALK_079571.50.85.x-R	*prn2-2* T-DNA line genotyping
TGCTGTCATCCACTGCAATAG	SAIL_1243_E02-L	*prn3* T-DNA line genotyping
TTTGGTAACATGCGCTACAAAC	SAIL_1243_E02-R	*prn3* T-DNA line genotyping
GGATGGTTCTCGAAAGAGGAC	SALK_138671.12.55.x-L	*prn4-1* T-DNA line genotyping
TTCGATAAGAAACATTTGCGG	SALK_138671.12.55.x-R	*prn4-1* T-DNA line genotyping
AGGTTGCTTTGTGAACCATTG	SALK_125909.33.10.x-L	*prn4-2* T-DNA line genotyping
CGTGAATTCTCAGAGTGGAGC	SALK_125909.33.10.x-R	*prn4-2* T-DNA line genotyping
ATATCCACGGGGTCCATTAAC	SALK_100855.51.75-LP	*prn4-3* T-DNA line genotyping
GTGCTTCCAAATGAAACCATG	SALK_100855.51.75-RP	*prn4-3* T-DNA line genotyping
AGCAGCTTGAGACTTCCAATG	GT19099-LP1	*prn4-4* T-DNA line genotyping
CATGGTTTCATTTGGAAGCAC	GT19099-RP1	*prn4-4* T-DNA line genotyping
TACGAATAAGAGCGTCCATTTTAGAGTGA	spm	for SM T-DNA lines genotyping
ATTTTGCCGATTTCGGAAC	LBb1.3	for SALK T-DNA lines genotyping
GCCTTTTCAGAAATGGATAAATAGCCTTGCTTCC	LB1	for SAIL T-DNA lines genotyping
GTTTTCATTTAGGTTTATCAGA	AtPRN2-1F	semi quantitative RT-PCR for PRN2
TCATTGTGACCTCCAATA	AtPRN2-1R	semi quantitative RT-PCR for PRN2
GGCGATGAAGCTCAATCCAAACG	actinF	For semi quantitative RT-PCR
GGTCACGACCAGCAAGATCAAGACG	actinR	For semi quantitative RT-PCR
TTCCTCGAACTCTTCGCCTATTC	AtPRN2-qPCR-F	For qPCR analysis of PRN2
CACACGCTAACACCATCATT	AtPRN2-qPCR-R	For qPCR analysis of PRN2
ATGGAGATTAACGGGGCACAC	AtPAL1-F	For qPCR analysis of PAL1 (At2g37040)
GTACCGCCGAGAACACCGCC	AtPAL1-R	For qPCR analysis of PAL1 (At2g37040)
GCAGAAGGGAGAAATCAACG	AtC4H-F	For qPCR analysis of C4H (At2g30490)
CAATATAATCCCGGGACAGC	AtC4H-R	For qPCR analysis of C4H (At2g30490)
GATTTGAGCTCGATAAGAGTGGTG	At4CL1-F	For qPCR analysis of 4CL1 (At1g51680)
ATTTGCTAGTTTTGCCCTCA	At4CL1-R	For qPCR analysis of 4CL1 (At1g51680)
CATTCACTCTTTCCCGCTTC	AtHCT-F	For qPCR analysis of HCT (At5g48930)
GTTCCCATCCTCCTTGGATT	AtHCT-R	For qPCR analysis of HCT (At5g48930)
CTCATTACGTGAAGGTGAGAAAAGT	AtC3H1-F	For qPCR analysis of C3H1 (At2g40890)
GATCTTGACATCTGCGTTGC	AtC3H1-R	For qPCR analysis of C3H1 (At2g40890)
GGCCCTGCTCTTCCCGTTC	AtCCoAOMT1-F	For qPCR analysis of CCoAOMT1 (At4g34050)
GGTGCATCAGGAGGAGCCA	AtCCoAOMT1-R	For qPCR analysis of CCoAOMT1 (At4g34050)
TGTGGATGTTCGCGATGTC	AtCCR1-F	For qPCR analysis of CCR1 (At1g15950)
GAGGAGCAAGATGGCCTTTC	AtCCR1-R	For qPCR analysis of CCR1 (At1g15950)
GTCATGCTCGACCGTATCCT	AtCOMT1-F	For qPCR analysis of COMT1 (At5g54160)
GCATCTTCGATGACATGTGG	AtCOMT1-R	For qPCR analysis of COMT1 (At5g54160)
GGTCCGGTCGGTCTCTTGTAA	AtF5H1-F	For qPCR analysis of F5H1 (At4g36220)
CACGAGCCGCTTGTTTATCC	AtF5H1-R	For qPCR analysis of F5H1 (At4g36220)
TGATCTTGGCATGTCTAATTACC	AtCAD5-F	For qPCR analysis of CAD5 (At4g34230)
GACATCAACGACGAACCTAT	AtCAD5-R	For qPCR analysis of CAD5 (At4g34230)
GGGGACAAGTTTGTACAAAAAAGCAGGCTTACACCACCCAAATAGCTTC	AtPRN2-1F	For construction of proAtPRN2::GUS transgenic plants
GGGGACCACTTTGTACAAGAAAGCTGGGTAATACTTTAAAGTCGTCTTCTCT	AtPRN2-1R	For construction of proAtPRN2::GUS transgenic plants
CCCAACTACAATAATGCAAC	VND6q-3F	For qPCR analysis of VND6 (At5g62380)
GCTCATGATTAGCTGAGAA	VND6q-3R	For qPCR analysis of VND6 (At5g62380)
GGACGAATAAAGATCAGAACGA	VND7q-F	For qPCR analysis of (At1g71930)
ATGCGGATGTATGACTTGTGTC	VND7q-R	For qPCR analysis of (At1g71930)
CAAGCTTGAGCCTTGGGATA	SND1q-5F1	For qPCR analysis of (At1g32770)
TGGTCCCGGTTGGATACTT	SND1q-5R1	For qPCR analysis of (At1g32770)
TCATGAAACTCCCAAACCTCGAAAG	NST1q-F	For qPCR analysis of (At2g46770)
TAACAAAGCTAGTGTCGACCACGTT	NST1q-R	For qPCR analysis of (At2g46770)
TCCCTAACCTCGAATGCCATAACAA	NST2q-F	For qPCR analysis of (At3g61910)
CCCATTTAACTGTGAGGCAACGAGC	NST2q-R	For qPCR analysis of (At3g61910)
GAATGTGAAGAAGGTGATTGGTACA	MYB46q-F	For qPCR analysis of (At5g12870)
CGAAGGAACCTCAGTGTTCATCA	MYB46q-R	For qPCR analysis of (At5g12870)

CTTTGTCATTCAGAAAGCTTCAAGG	MYB83q-F	For qPCR analysis of (At3g08500)
ATCGACTTGGAAATCAAGGAAGGGAAA	MYB83q-R	For qPCR analysis of (At3g08500)
CCAGAGAACAGAGCTCTTCAAGAG	MYB58q-F	For qPCR analysis of (At1g16490)
ATGTATGAGGAGCTCGTAACTCTC	MYB58q-R	For qPCR analysis of (At1g16490)
GAAGAAGAGTCTGATGAGGATGAGG	MYB63q-F	For qPCR analysis of (At1g79180)
CATGAGCTCGTAGTTCTTCAAGAGTG	MYB63q-R	For qPCR analysis of (At1g79180)
GGTGTTGGTGGAACATTATATGACC	MYB85q-F	For qPCR analysis of (At4g22680)
GGTGAACAGTCAAAACCCAAAATC	MYB85q-R	For qPCR analysis of (At4g22680)
GATCAATGGGACGATTCACAACAAT	MYB103q-F	For qPCR analysis of (At1g63910)
AAACGAAGAAGGGAAAGAAGAAGATAA	MYB103q-R	For qPCR analysis of (At1g63910)
TAATACCTCAAATTTCTTCGACAATGGC	MYB61q-F	For qPCR analysis of (At1g09540)
CGGTGTGTTCAAATACTCTGACCAT	MYB61q-R	For qPCR analysis of (At1g09540)
TAACGTGGCCAAAATGATGC	PDF2-F	housekeeping gene (At1g13320) for qPCR
GTTCTCCACAACCGCTTGGT	PDF2-R	housekeeping gene (At1g13320) for qPCR
AACTTTGGTGGTTTGTGTTTTGG	UBQ10-F	housekeeping gene (AT4G05320) for qPCR
TCGACTTGTCATTAGAAAGAAAGAGATAA	UBQ10-R	housekeeping gene (AT4G05320) for qPCR
GAAAAATCTTAGAAAGCAGCAAAG	PttPRN2-F	For construction of proPttPRN2::GUS transgenic plants
ATACTACGAGCAATCAAAAAGATAC	PttPRN2-R	For construction of proPttPRN2::GUS transgenic plants
GGTTTCAGCACACCCAGATT	PttPRN2q-F	For qPCR analysis of (Potri.002G231900)
GAACTTCAGCTCGCTCCTTC	PttPRN2q-R	For qPCR analysis of (Potri.002G231900)

References

Bollhöner B, Zhang B, Stael S, Denancé N, Overmyer K, Goffner D, Van Breusegem F, Tuominen H. 2013. Post mortem function of AtMC9 in xylem vessel elements. *New Phytologist* **200:** 498-510.

De Meester B, de Vries L, Özparpucu M, Gierlinger N, Corneillie S, Pallidis A, Goeminne, G, Morreel K, De Bruyne M, De Rycke R, Vanholme R, Boerjan W. 2018. Vessel-specific reintroduction of CINNAMOYL-COA REDUCTASE1 (CCR1) in dwarfed *ccr1* mutants restores vessel and xylary fiber integrity and increases biomass. *Plant Physiology* **176:** 611-633.

Edgar, RC 2004. MUSCLE: multiple sequence alignment with high accuracy and high throughput. *Nucleic Acids Research* **32:** 1792-1797.

Guindon S, Dufayard JF, Lefort V, Anisimova M, Hordijk W, Gascuel O. 2010. New algorithms and methods to estimate maximum-likelihood phylogenies: assessing the performance of PhyML 3.0. *Systematic Biology* **59:** 307-321.

Tamura K, Peterson D, Peterson N, Stecher G, Nei M, Kumar S. 2011. MEGA5: Molecular Evolutionary Genetics Analysis using maximum likelihood, evolutionary distance, and maximum parsimony methods. *Molecular Biology and Evolution* **28:** 2731-2739.

Zhang B, Tremousaygue D, Denancé N, van Esse HP, Hörger AC, Dabos P, Goffner D, Thomma BPHJ, van der Hoorn RAL, Tuominen H. 2014. PIRIN2 stabilizes cysteine protease XCP2 and increases susceptibility to the vascular pathogen Ralstonia solanacearum in Arabidopsis. *Plant Journal* **79:** 1009-1019.
